# Bias-induced conductance switching in single molecule junctions containing a redox-active transition metal complex

**DOI:** 10.1007/s00706-016-1795-6

**Published:** 2016-08-15

**Authors:** Georg Kastlunger, Robert Stadler

**Affiliations:** Institute of Theoretical Physics, Vienna University of Technology, TU Wien, Vienna, Austria

**Keywords:** Electron transfer, Single molecule electronics, Density functional theory, Marcus theory, Redox reactions, Nanostructures

## Abstract

**Abstract:**

The paper provides a comprehensive theoretical description of electron transport through transition metal complexes in single molecule junctions, where the main focus is on an analysis of the structural parameters responsible for bias-induced conductance switching as found in recent experiments, where an interpretation was provided by our simulations. The switching could be theoretically explained by a two-channel model combining coherent electron transport and electron hopping, where the underlying mechanism could be identified as a charging of the molecule in the junction made possible by the presence of a localized electronic state on the transition metal center. In this article, we present a framework for the description of an electron hopping-based switching process within the semi-classical Marcus–Hush theory, where all relevant quantities are calculated on the basis of density functional theory (DFT). Additionally, structural aspects of the junction and their respective importance for the occurrence of irreversible switching are discussed.

**Graphical abstract:**

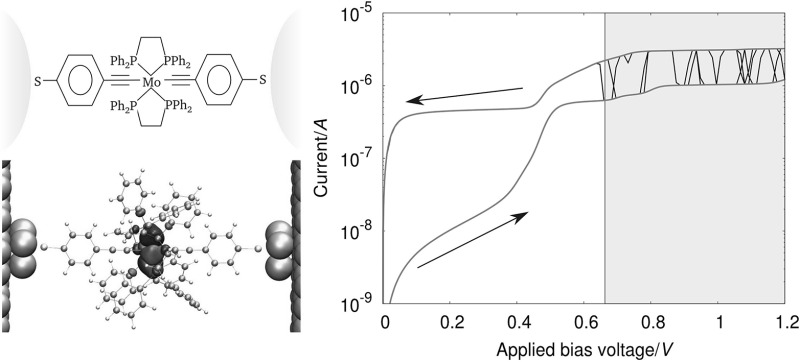

## Introduction

Single molecule electronics (SME) provides a promising alternative to conventional semiconductor electronics, where it is envisioned that single, or small ensembles of molecules could be applied as active or passive building blocks in electronic circuits [2, 3].

A variety of possible applications for molecular components in electronic circuits could be identified in the past decades. Single molecules were proposed to function as both passive (wires) [4–13] and active (diodes, transistors, switches) [14–31] devices in electronic components, where their most significant benefit is that their intrinsic functionality can be designed reliably by means of chemical synthesis.

Single molecule switching mechanisms are based on either conformational changes triggered by photons [14–17] or bias [18–22, 31, 32], spin crossover [24–26] or a redox reaction, which is performed via the introduction of oxidizing or reducing agents [27] or a gate electrode in an electrochemical cell [28–30].

The possible applicability of transition metal complexes for single molecule switches was first investigated by the group of Jens Ulstrup [33–36] showing significant redox switching potential of such compounds within the junction supported by a good alignment of the molecular eigenstates with the electrodes Fermi energy. An electron transfer kinetics model was derived for the explanation of the trends found in these measurements [34–37], which described the electron transport in such junctions as a two-step process of subsequent resonant tunneling events aided by the vibrational relaxation of the molecular orbitals. An adaptation of this scheme based on electron hopping in terms of Marcus theory for single molecule junctions has been addressed by Ulstrup and Kuznetsov [38, 39] and has been further developed by Nitzan and co-workers recently [40]. Both groups, however, did not address the determination of the key parameters in a junction environment from density functional theory (DFT) calculations, but rather used model systems, such as rigid spheres between two metallic electrodes.

Migliore and Nitzan have recently proposed an explanation for hysteresis in single molecule *I*/*V* measurements based on the interplay of coherent tunneling, defining the conductance of the junction, and electron hopping causing a time delay or hysteresis in the *I*/*V* curves [41, 42]. The most important ingredient of this model is a localized state on the compound exhibiting a low degree of electronic coupling to the electrodes. Based on this model in combination with DFT calculations, hysteresis effects found in mechanically controlled break junction (MCBJ) experiments performed by Schwarz et al. [1] have been analyzed by us theoretically. In this work, three transition metal complexes with a Fe, Ru, and Mo-center, respectively, have been studied regarding their electronic ground state and switching properties, where the derivation of the structure-dependent key parameters for Migliore and Nitzan’s 2-channel scheme for these structures from DFT has been achieved.

This paper tries to move further in this direction with a special emphasis set on a more detailed analysis of the key quantities relevant for the occurrence of conductance switching in transition metal complex-based single molecule junctions and their relation to the structural properties of the respective compound.

## Results and discussion

While the description of coherent electron transport in single molecule junctions is already well established, a treatment of incoherent sequential electron hopping in the literature is still mostly limited to intramolecular charge transfer in push–pull molecules or the charging of adsorbed molecules on a single surface as proposed in a series of articles by Rudolph Marcus [43–45].

In its original formulation, Marcus’ theory proposed a mechanism for electron transport reactions in a solvent environment, which is driven by the nuclear relaxation of both the molecular charge carriers and the solvent. Based on this proposition, Marcus’ derived the definition for the classical Gibbs activation energy for such a process as [43]

1 $${\Delta G_{\text{act}} = \frac{{\left( {\lambda + \Delta G^{0} } \right)^{2} }}{4\lambda }},$$

where *λ*, the so-called reorganization energy, represents the energy barrier arising from the need of both reactant and solvent atoms to adapt to the resulting charge distribution in the products and Δ*G*^0^ is the Gibbs free energy for the reaction. Using this definition of Δ*G*_act_, a formulation for the electron transfer rate (*k*_ET_) could be established, which has an Arrhenius type form:

2 $${k_{\text{ET}} = Ae^{{\frac{{ - \left( {\lambda + \Delta G^{0} } \right)^{2} }}{{4\lambda k_{b} T}}}} }.$$

The classical definition in the exponent of Eq. (2) is in principle, only valid in the high temperature limit, where nuclear tunneling through the barrier created by the activation Gibbs free energy can be neglected, which also applies for lower temperatures when the energy barrier of the reaction is directly dependent on an applied bias as it is the case for the remainder of our article. In the non-adiabatic case of semi-classical Marcus–Hush theory, the pre-exponential factor *A* in Eq. (2) is defined as the rate of electron transfer for the system at the transition point [43, 46, 47]

3 $${A = \frac{2\pi }{\hbar }\frac{{H_{\text{DA}}^{2} }}{{\sqrt {4\pi \lambda k_{b} T} }}},$$

where *H*_DA_ = <D|H|A> is the matrix element in the Hamiltonian *H* between the donor (D) and acceptor (A) states, which are the initial and final states for the transferred charge, respectively.

When describing electron transfer reactions for an atom or molecule adsorbed on electrodes, Marcus’ original picture of a reaction driven by thermally induced vibrations has to be modified, because here the activation free energy is dependent on an applied bias or potential. This potential shift now allows the reactants to reach the transition point in contrast to an activation by temperature-induced vibrations. For the definition of *k*_ET_ in such a molecule–electrode setup, it is crucial to account for the large number of electronic bands near the metal’s Fermi level *μ*. Therefore, *k*_ET_ has to be adapted by including the various surface states able to act as donor or acceptor states, thereby changing the picture of the two intersecting parabolas to multiples of them [43, 44], where each of the parabolas describes one reactant/product pair consisting of the molecular orbital (MO) localized on the adsorbed molecule, which is relevant for the reaction and an individual metal electronic state out of the manifold. As a consequence, the Gauss-like expression in Eq. (2) has to be replaced by an error function, accounting for all metal bands which can participate in the reaction [40, 48, 49]. For the respective reduction and oxidation reactions, the corresponding reaction rates can then be expressed as:

4 $${k_{\text{rd}} = \frac{2\pi }{\hbar }\frac{{H_{\text{DA}}^{2} }}{{\sqrt {4\pi \lambda k_{b} T} }}\int {e^{{ - \frac{{\left( {\lambda - \Delta G^{0} + e\Phi - E} \right)^{2} }}{{4\lambda k_{b} T}}}} } f\left( E \right){\text{d}}E,}$$

and

5 $${k_{\text{ox}} = \frac{2\pi }{\hbar }\frac{{H_{\text{DA}}^{2} }}{{\sqrt {4\pi \lambda k_{b} T} }}\int {e^{{ - \frac{{\left( {\lambda + \Delta G^{0} - e\Phi + E} \right)^{2} }}{{4\lambda k_{b} T}}}} } \left[ {1 - f\left( E \right)} \right]{\text{d}}E,}$$

where *eΦ* represents the change in *μ* due to the applied external potential, and *f*(*E*) is the Fermi–Dirac distribution of the electrode.

**Fig. 1:**
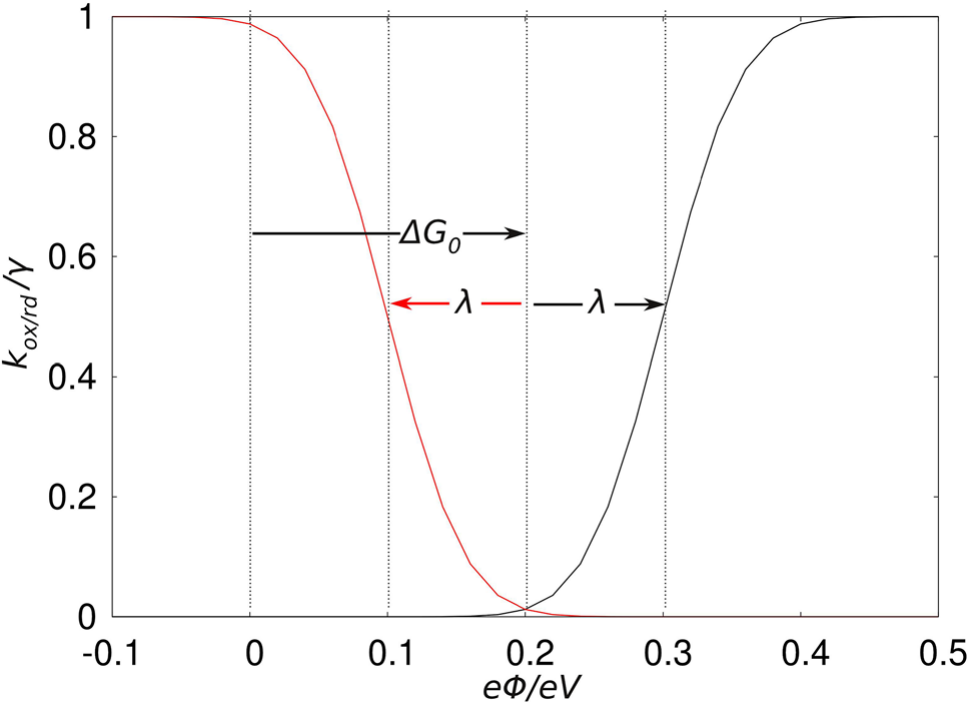
Electron transfer rates of a reaction between a molecular compound and a metal electrode in dependence on the applied bias, with *k*_ox_ shown in *black* and *k*_rd_ in *red*. The ground state of the molecule was chosen to be its reduced form. The rates have been normalized by excluding the preexponential factor *γ* = 2*π*/*ħ* * *H*_DA_^2^ in Eqs. (4) and (5). We used the parameters: Δ*G* ^0^ = 0.2 eV, *λ* = 0.1 eV, *T* = 50 K

Figure 1 shows how an applied external potential or bias voltage influences the reaction rates at a molecule–electrode interface. As a consequence of their functional forms in Eqs. (4) and (5), the transfer rates behave like error functions. The error functions for the two types of reaction cross each other at Δ*G*^0^ and their inflection point is shifted by ±*λ* with respect to this crossing. The mirror symmetry of these two error functions with respect to each other arises from their respective Fermi–Dirac distributions, which determine whether the occupied or unoccupied metal bands participate in the reaction and their assumed symmetry in the density of states (DOS) close to *μ*. The influence of the electronic coupling was neglected in Fig. 1 by normalizing the super exchange rate *γ* = 2*π*/*ħ* * *H*_DA_^2^ to one, which in real systems is a scaling factor for the reaction rates.

Moving from the molecule/single electrode, charge transfer occurring in an electrochemical cell to a single molecule junction setup, where the molecule is connected to two metallic electrodes and, therefore, the electron or hole can, in principle, take place in both directions. Therefore, for each of the two metallic surfaces, separate reaction rates have to be defined for both kinds of reaction [42]:

6 $${k_{{{\text{ox}},{\text{K}}}} \left( V \right) = \frac{2\pi }{\hbar }\frac{{H_{\alpha ,K}^{2} }}{{\sqrt {4\pi \lambda k_{\text{b}} T} }}\int {{e}^{{ - \frac{{\left( {\lambda + \Delta G_{\alpha }^{0} + E + e\varPhi_{K} } \right)^{2} }}{{4\lambda k_{b} T}}}} } \left[ {1 - f\left( E \right)} \right]{\text{d}}E}$$

and

7 $$\begin{aligned}{{k_{{\rm{rd,K}}}}\left( V \right) = \frac{{2\pi }}{\hbar}\frac{{H_{\alpha, {\rm{K}}}^2}}{{\sqrt {4\pi \lambda {k_b}T} }}\int {{e^{ - \frac{{{{\left( {\lambda - \Delta G_\alpha ^0 - E + e{\Phi _{\rm{K}}}} \right)}^2}}}{{4\lambda {k_b}T}}}}} f\left( E \right){\rm{d}}E}\end{aligned}$$

with *Φ*_K_ = *∓ V*/*2* and *K* referring to the left (*L*) and right (*R*) electrode.

For such a setup, one could, in principle, calculate Δ*G*^0^ by comparing the ionization potential of the compound in its neutral and charged state and relate it to the work function of the metal electrodes in both states. Such a procedure, however, would not include screening effects and charge transfer during the adsorption process, which lead to a shift of the molecular eigenstate energies relative to *μ* and a reduction of the HOMO–LUMO gap size. Therefore, we decided to apply a method which includes these effects directly and is also consistent with our single particle description of coherent tunneling by defining

8 $${\Delta G_{\alpha }^{0} = \left( {\varepsilon_{\alpha ,1} - \mu_{1} } \right) - \left( {\varepsilon_{\alpha ,0} - \mu_{0} } \right)},$$

where *ε*_*α*,0_ − *μ*_0_ represents the MO energy of the eigenstate participating in the electron exchange reaction in the initial state of the system relative to the metal Fermi level *μ*_0_, and *ε*_*α,*1_ − *μ*_1_ is the energy of the MO after the redox reaction has happened with the electrodes’ Fermi level after the reaction *μ*_1_ as its reference. Since no substantial change in the Fermi level of a semi-infinite metal electrode results from the addition or subtraction of a single electron, one can simplify Eq. (8) to

9 $${\Delta G_{\alpha }^{0} \approx \varepsilon_{\alpha ,1} - \varepsilon_{\alpha ,0} }$$

in a good approximation.

In a single molecule junction setup, an additional contribution has to be added to the definition of the reorganization energy compared to Marcus’ and Hush’s original definition [50, 51], namely the difference in screening of the charge by the metallic surfaces before and after the reaction, commonly described in terms of an image charge model [38, 39, 43]. As a result, *λ* is now defined as a sum of three contributions, namely

10 $${\lambda = \lambda_{\text{in}} + \lambda_{\text{solv}} + \lambda_{\text{img}} }.$$

The measurements which will be interpreted theoretically later in this paper, however, have been performed in UHV, where *λ*_solv_ = 0; therefore, we would like to refer to an earlier paper [52] for our definition of *λ*_solv_ in an electrochemical environment.

The calculation of *λ*_in_ is straightforward, since it is the energy, which is required to relax the nuclei of the reactants from their energetic minimum at the systems equilibrium geometry, namely the uncharged molecule in the junction setup, to their optimal configuration in its final state, i.e., the charged compound between the surfaces after the charge has been transferred. Because during the reaction no significant structural rearrangement of the infinitely large metal electrodes takes place, only changes in the molecular geometry have to be considered for *λ*_in_. Therefore, *λ*_in_ for a redox reaction in a single molecule junction has been calculated from the difference of the neutral (initial, *i*) molecule’s total energies in the equilibrium structure of its charged (final, *f*) state *E*_0_(*R*_*f*_) and its initial geometry *E*_0_(*R*_*i*_).

For the second reaction, i.e., the subsequent reduction, *λ*_in,fi_ is defined accordingly, namely as the difference of the total energies of the charged system in the nuclear arrangement of the neutral system *E*_1_(*R*_*i*_) and in that of its own equilibrium geometry *E*_1_(*R*_*f*_). According to Marcus theory, the curvature of both corresponding Gibbs free energy parabolas should be identical. In numerical calculations, however, the respective values can differ slightly, which is why we used an average of the two definitions for the calculation of the inner part of the reorganization energy [53]:

11 $${\lambda_{\text{in}} = \frac{{\lambda_{\text{in,if}} + \lambda_{\text{in,fi}} }}{2} = \frac{{E_{0} \left( {R_{f} } \right) + E_{ + 1} \left( {R_{i} } \right) - E_{0} \left( {R_{i} } \right) - E_{ + 1} \left( {R_{f} } \right)}}{2}}.$$

The final contribution to *λ* in Eq. (10), namely *λ*_img_, was calculated from an image charge model where an infinite sum of Coulomb interactions arises from the partially charged molecule’s infinite number of mirror images in the 2-electrode setup [54, 55]

12 $$\begin{array}{*{20}c} {\lambda_{\text{img}} = \frac{ - 1}{{8\pi \epsilon_{0} }}\varSigma_{i}^{N} \varSigma_{j}^{N} \Delta q_{i} \Delta q_{j} \times \varSigma_{n = 1}^{\infty } } \\ {\left\{ {\begin{array}{*{20}c} {\frac{1}{{\sqrt {\left( {z_{i} + z_{j} - 2nL} \right)^{2} + R_{ij}^{2} } }} + \frac{1}{{\sqrt {\left( {z_{i} + z_{j} + 2\left( {n - 1} \right)L} \right)^{2} + R_{ij}^{2} } }}} \\ { - \frac{1}{{\sqrt {\left( {z_{i} - z_{j} + 2nL} \right)^{2} + R_{ij}^{2} } }} - \frac{1}{{\sqrt {\left( {z_{i} - z_{j} - 2nL} \right)^{2} + R_{ij}^{2} } }}} \\ \end{array} } \right\}} \\ \end{array}$$

with $R_{ij}^{2}$ = (*x*_*i*_ − *x*_*j*_)^*2*^ + (*y*_*i*_ − *y*_*j*_)^*2*^ and *x*_*i,j*_, *y*_*i,j*_, *z*_*i,j*_ the positions of the atoms of the molecule, with the z coordinate being the transport direction, while Δ*q*_*i*/*j*_ corresponds to the changes in atomic partial charges caused by the reaction, which have been determined as differences for the neutral and charged states of the molecule from a Mulliken charge analysis [56].

The transfer integral *H*_*α,*K_ (*K* ϵ {*L*,*R*}) in Eqs. (6) and (7), as the third key parameter in a description of electron hopping within Marcus theory, is in principle both energy and ***k***-point dependent when surfaces are involved, because it is related to the density of states of the metal electrodes *ρ*(*ε*):

13 $${H_{{\alpha ,{\text{K}}}}^{2} \left( {E,\vec{k}} \right) = H_{{\alpha ,{\text{i}}}}^{2} \left( k \right)\rho \left( {E,\vec{k}} \right)},$$

where *H*_*α,*i_(***k***) is the electronic coupling for the molecular eigenstate α and the metallic band i in the electrode *K* at each energy *E* and ***k***-point ***k*** [40, 42]. For the analysis presented in this paper, we use a simplified scheme on the basis of Landauer theory as it was already introduced in earlier publications [52, 57] for the evaluation of *H*_*α,*K_. This scheme exploits the fact that the width of a peak in the single MO transmission function calculated within Landauer theory is directly related to *H*_*α,*K_ on a single particle level. Accordingly, for a given molecular orbital *α*, *H*_*α,*L_ and *H*_*α,*R_ has been computed from the width of the resulting single channel transmission peak, which in such a case has the Lorentzian type form:

14 $${T_{\alpha } \left( E \right) = \frac{{ 4 {\text{H}}_{{\alpha ,{\text{L}}}} H_{{\alpha ,{\text{R}}}} }}{{\left( {H_{{\alpha ,{\text{L}}}} + H_{{\alpha ,{\text{R}}}} } \right)^{ 2} + \left( {E - \varepsilon_{\alpha } } \right)^{ 2} }}}.$$

**Fig. 2:**
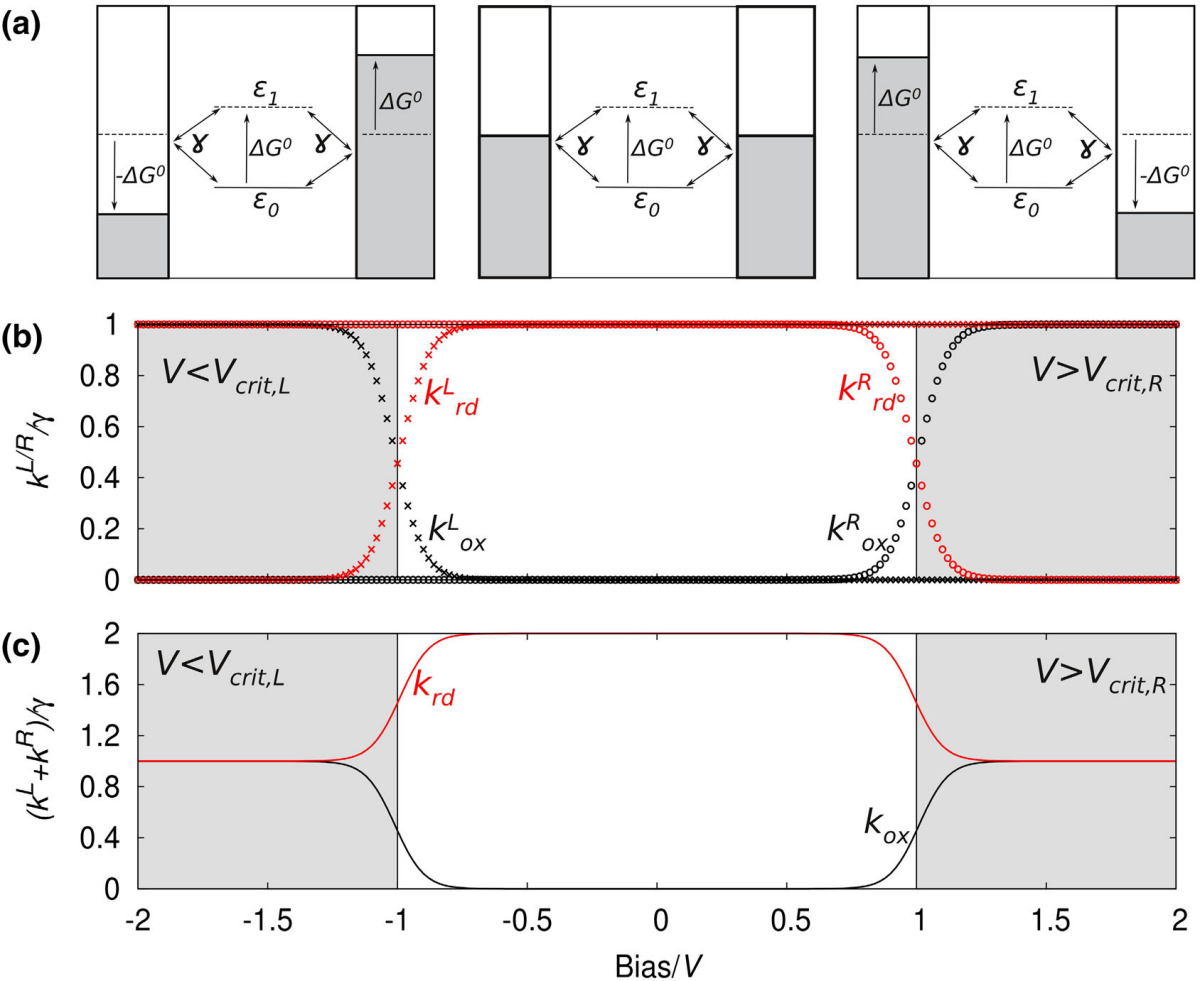
Schematic representation of a model system consisting of a single molecular eigenstate between two metal electrodes and its response to an applied bias voltage (**a**), where in **b** the bias dependence of the rates at the *left* and *right* electrodes is shown as *stars* and *circles*, and the *k*_ox_,_K_ and *k*_rd_,_K_ are shown in *red* and *black*, respectively. Finally, in **c** the total transfer rates for the reaction happening at any of the two molecule–electrode interfaces, *k*_ox_ and *k*_rd,_ are shown. A symmetric coupling to both electrodes was chosen and the reaction rates have been normalized by setting *γ* = 1 or in other words by plotting *k*/*γ* in a symmetrically coupled junction system

Figure 2 shows a schematic picture of the bias dependence of the reaction rates introduced in Eqs. (6)–(7) explicitly as well as the total reaction rates for oxidation and reduction, respectively, for the case of *λ* − >0 and *H*_*α,*L_ = *H*_*α,*R_. The model system chosen for this figure consists of a single electron’s eigenstate *α* localized on the molecule with an eigenvalue *ε*_*0*_ symmetrically coupled to two metallic electrodes. For *ε*_0_ − *μ* < 0, *α* is occupied in the ground state of the system, in the absence of any bias voltage. A source-drain bias can now be applied by a shift of the electrodes’ Fermi levels in Eqs. (6)–(7) and Fig. 2, resulting in *μ*_L_(*V*) = *μ*_L,0_ + *eV*/2 and *μ*_R_(*V*) = *μ*_R,0_ − *eV*/2, respectively. When the critical voltage of

15 $${\begin{aligned} V_{\text{crit,ox,L}} = \frac{2}{e}\left( { - \Delta G^{0} - \lambda } \right) \\ V_{\text{crit,ox,R}} = \frac{2}{e}\left( {\Delta G^{0} + \lambda } \right) \\ \end{aligned} }$$

is approached, the activation energy for an electron hop from the molecule to the electrodes is reduced until it reaches a value of zero at *V*_crit_ [40]. From this point on, the jump of an electron from the molecule to the respective electrode combined with a structural relaxation to shifts *α*’s eigenenergy from *ε*_0_ to *ε*_1_ leads to a reduction of the systems total energy, which is the systems new ground state at the respective applied bias. The time scale for one specific electron jump in this bias region is defined by *γ*^−1^.

In panel b of Fig. 2 the transition into this barrierless bias regime is visible as an increase in *k*_ox,K_ (black curves in Fig. 2) until the inflection point of the error function is reached at *V*_crit_, where *k*_ox,K_ reaches half of its maximum value.

For the reduction reaction starting from the orbital *α* in its unoccupied state with an eigenenergy *ε*_1_, the situation is reversed. For *λ* < Δ*G*^0^, *k*_rd,K_ is at its maximum, when no bias is applied, meaning that there is no barrier for the electron exchange reaction to either of the two electrodes. When the bias is applied, however, *k*_rd_ can be reduced, which is due to the fact that either *μ*_L_ or *μ*_R_ (depending on the sign of the bias) is lowered in energy. In contrast to *k*_ox_, *k*_rd,K_ now falls to zero, when the bias passes the critical voltages

16 $${\begin{aligned} V_{\text{crit,rd,L}} = \frac{2}{e}\left( { - \Delta G^{0} + \lambda } \right) \\ V_{\text{crit,rd,R}} = \frac{2}{e}\left( {\Delta G^{0} - \lambda } \right) \\ \end{aligned} }$$

(We note that for *λ* − >0 *V*_crit,ox,K_ = *V*_crit,rd,K_, which is the case depicted in Fig. 2.)

When the participation of both electrodes is taken into account, the respective reaction rates describing the redox reaction with one electrode and the molecule simply add up. This is due to the fact that no matter in which direction the electron(hole) exchange happens, it always results in a reduction or oxidation of the molecular species. These summed up reaction rates *k*_rd_ and *k*_ox_ are shown in panel c of Fig. 2. The total oxidation rate in the described case is still zero at small biases, since no oxidation reaction of the molecule, with an electron moving to any of the two electrodes happens. For *V* < *V*_crit,ox,L_ and *V* > *V*_crit,ox,R_, however, a reaction involving one of the two leads happens with a frequency of *γ*_K_, while the opposite electrode does not participate to the same reaction. This is due to the fact that at *V*_crit,L/R_ the energy barrier for the electron (or hole) transfer from the molecule to *L*/*R* is fully compensated by the applied bias, while on the other electrode (*R*/*L*) it is even increased due to the relation *Φ*_L_ = −*Φ*_R_.

The total reduction rate *k*_rd_ for our model system, in panel c of Fig. 2, on the other hand, is *γ*_L_ + *γ*_R_, (or *2γ* in a symmetric junction) for zero bias, which is due to the systems total energy reduction, when an electron occupying an electrode surface state at *μ* is transferred onto *α* with its eigenenergy *ε*_0_. When no external potential is applied, the energy gain is the same on both electrodes, because *μ*_L_ = *μ*_R_.

For *V* < *V*_crit,rd,L_ or *V* > *V*_crit,rd,R_, however, the reaction with one of the two respective electrodes becomes unfavorable, since its Fermi energy is lowered by such an amount that the occupation of *α* with an electron arriving from the respective electrode does not lead to a total energy reduction anymore, therefore, leading to *k*_rd,K_ *→* *0*. However, *k*_rd_ does never reach a value of zero since the reaction between the molecule and the other of the two electrodes is still barrierless and this electrode is, therefore, able to provide the electron for the reduction reaction.

Recently, Migliore and Nitzan [41] proposed a model mechanism causing hysteresis in *I*/*V* curves based on two different types of electron transfer reactions occurring simultaneously but on different time scales. While the faster one of the two reactions in this two-channel model is defining the measured conductance, the slower one is the reason for the hysteresis or switching observed in *I*/*V* measurements. In a single molecule junction setup, this means that coherent electron transport is mainly responsible for the conductance and defines the “fast channel”. For the switching in conductance for such compounds as described in this article, the most plausible mechanism is a change in the compounds redox state via electron hopping from one of the electrodes onto a localized eigenstate close to the electrodes’ Fermi level. This process can be quantitatively described in terms of electron transfer rates according to Marcus theory, as described above, where the key parameters are derived from DFT calculations.

Based on Migliore and Nitzan’s model, an algorithm for the simulation of such hysteresis effects and switching has been used for the theoretical analysis of the experimentally found bias driven switching found in Ref. [1], which we recapitulate in more detail in the following:

For this scheme, two different *I*/*V* curves are needed, one corresponding to the system before and one to the system after the redox reaction has occurred, i.e., for junctions with the molecule in its oxidized and reduced state, respectively. These two curves are the outer borders for the *I*/*V* curves measured in the experiments. In our simulations, the reduced (charged) state of the molecule in the junction was obtained following our earlier work [58], where we use a Cl atom to extract an electron from the molecule in the junction. Due to the inversion symmetry of the investigated compounds, only a second-order Stark effect arises from an applied electric field and, therefore, the *I*/*V* curves for both redox states of the setup can be approximated as:

17 $${I = G_{0} \int T \left( E \right)\left( {f_{\text{L}} - f_{\text{R}} } \right){\text{d}}E = G_{0} \int T \left( E \right){\text{d}}E}$$

where in this rigid band approach the bias *V* is replaced by the transmission function’s dependence on the electrons’ incident energy *E* at zero bias and polarization effects due to a finite bias are neglected.

In a next step, the hopping reaction involving a weakly coupled MO has to be analyzed regarding its time scale, to determine if and how often the corresponding redox reaction happens within the time span for the measurement of one individual current value in the experiment.

In our model, the system was only allowed to reside in one of the two redox states at any given point in time with no statistical averaging, which corresponds to the simulation of individual sweeps in the measurements of our experimental partners. Hence, we have to define a probability *P*(*V*) for a redox state to change for any given value of the applied voltage. For that purpose, two types of time intervals are defined, namely Δ*t*, the experimental integration time used for obtaining the current for each individual point in the *I*/*V* curves, and d*t*, which is a mere convergence parameter in our simulations, and which defines the time the system has for a single redox reaction, where both intervals are linked by the relation

18 $${\Delta t = n{\text{d}}t}$$

with *n* as the number of redox reactions which can maximally occur during Δ*t*.

Within each interval Δ*t*, the applied voltage *V* is constant and so are as a consequence *k*_ox/rd_(*V*), resulting in *P*(*V*) being defined by the simple products

19 $${\begin{aligned} P_{\text{ox}} \left( V \right) = k_{\text{ox}} {\text{d}}t \\ P_{\text{rd}} \left( V \right) = k_{\text{rd}} {\text{d}}t \\ \end{aligned} }$$

where either *P*_ox_(*V*) or *P*_rd_(*V*) is used in the simulation depending on the redox state of the compound at the beginning of each time interval d*t*.

At this point, the stochastic nature of the approach becomes important. From its definition in Eq. (19), *P*(*V*) could in principle have any values between 0 and infinity. Therefore, in order to define it as a proper probability with values between 0 and 1, one has to adjust d*t* or *n* accordingly, which does not qualitatively change the result. This is due to the inverse proportionality of d*t* and *n*, defined by Eq. (18), where a reduced time interval d*t*, lowers the probability of a single electron transfer reaction, but the corresponding increase in *n* increases the number of maximally possible reactions over the larger interval Δ*t* [1]. This probability *P*(*V*) is then compared with a random number *x* ranging from 0 to 1 to decide if a reaction takes place, allowing it only for *P*(*V*) > *x*. Finally, the current measured at each experimental bias point *V* within its respective time interval Δ*t* can be obtained from our simulations by

20 $${I\left( V \right)_{\Delta t} = \frac{1}{n}\varSigma_{i = 1}^{n} I\left( {V,s_{i} } \right)}$$

with

21 $${I\left( {V,s_{i} } \right) = \left( {1 - s_{i} } \right)I_{\text{ox}} \left( V \right) + s_{i} I_{\text{rd}} \left( V \right)}$$

where *s*_*i*_ ϵ {0,1} represents the redox state the system is in at the end of each d*t* window.

**Fig. 3:**
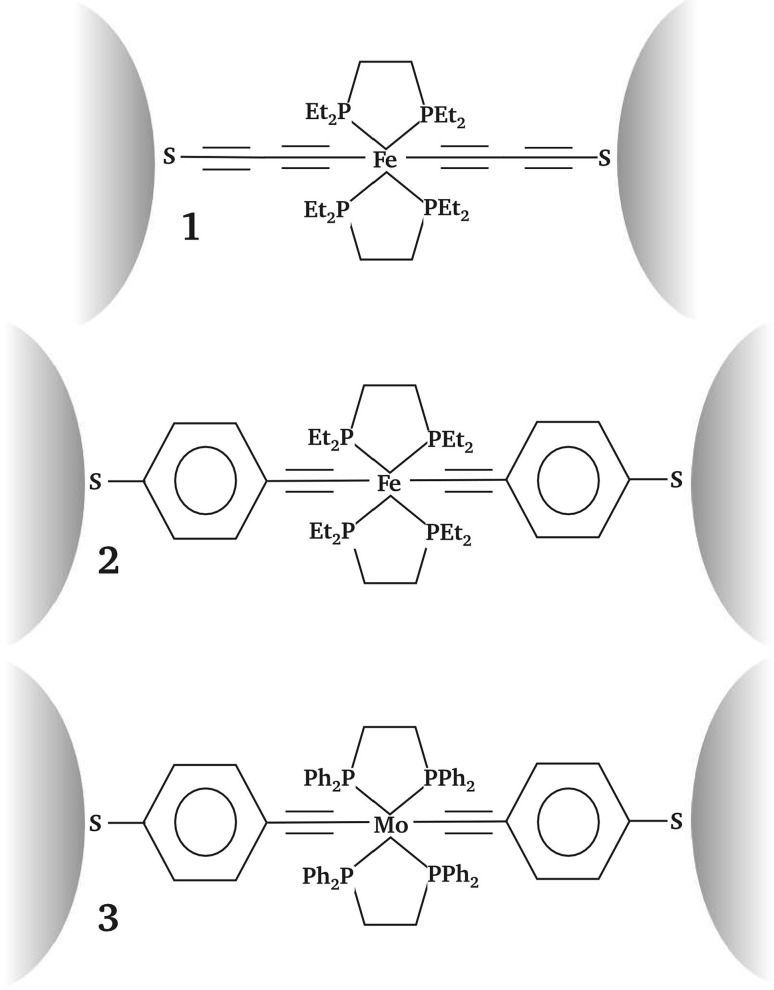
Molecular structures for the compounds **1**–**3** which we studied in this article in a junction setup

Let us now move on to DFT-based calculations for real single molecule junctions, namely *trans*-(SC_4_)_2_Fe(1,2-bis(diethylphosphino)ethane)_2_ (**1**), *trans*-(SC_6_H_4_–C≡C–)_2_Fe(1,2-bis(diethylphosphino)ethane)_2_ (**2**), and *trans*-(SC_6_H_4_–C≡C–)_2_Mo(1,2-bis(diphenylphosphino)ethane)_2_ (**3**), where we show their molecular structures in Fig. 3. These three particular compounds have been chosen to emphasize the influence of structural parameters for different molecules on their respective ability to produce hysteresis in STM or MCBJ based *I*/*V* measurements. From **1** to **2**, the effect of a change in the molecular anchor groups and, therefore, the preexponential factor *γ* in Eq. (2) defined by the transfer integral *H*_DA_ in Eq. (3) is studied. In the transition from **2** to **3**, the influence of the central metal atom, defining $\Delta G_{\alpha }^{0}$ in Eqs. (8) and (9) via the characteristic eigenenergy *ε*_*α*_ of a localized state *α*, is investigated. In such (strongly coupled) systems, the conductance through the junction is defined by coherent tunneling of electrons through the junction, which is well described by Landauer-Büttiker theory [59, 60].

**Fig. 4:**
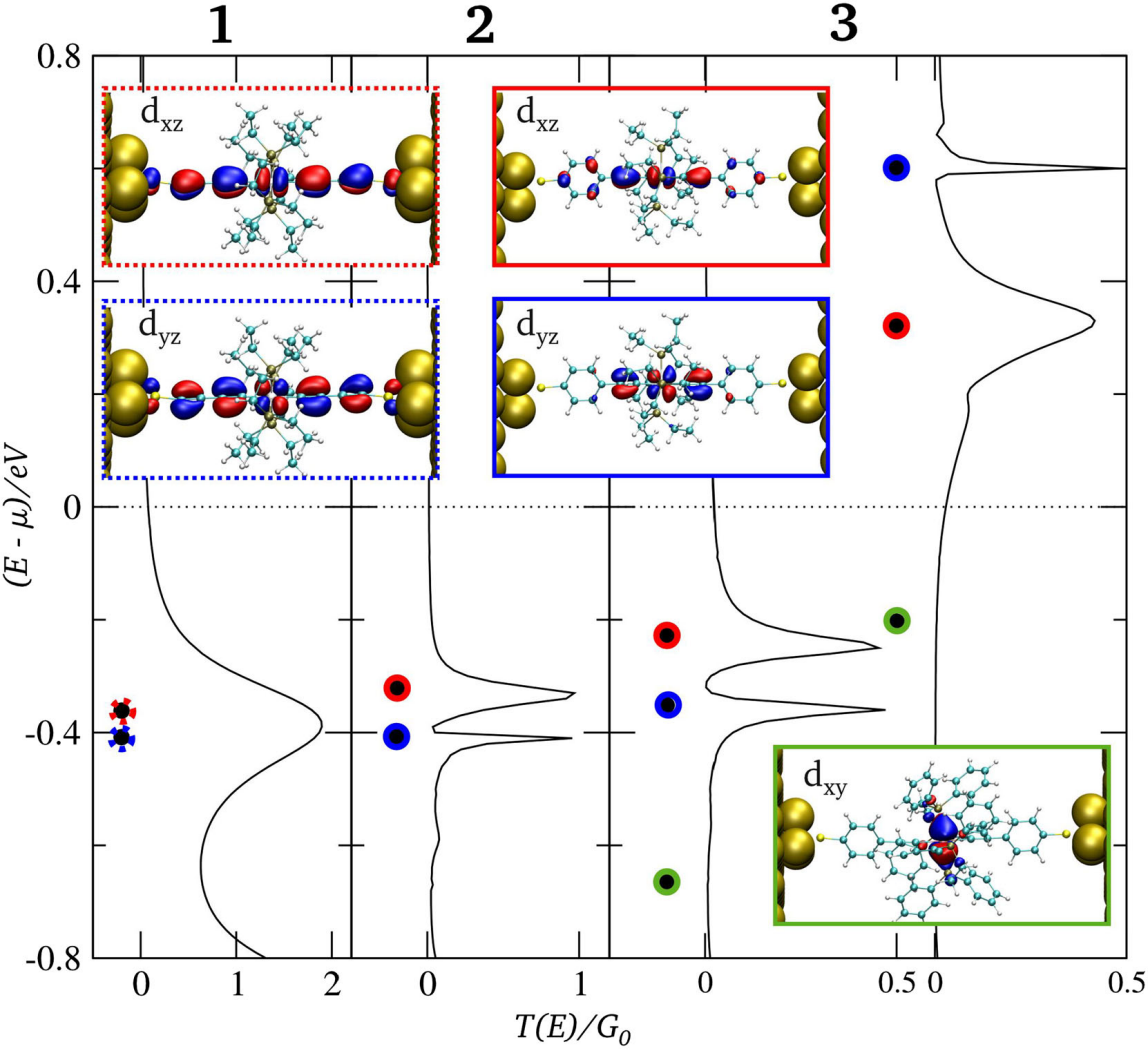
Transmission functions and MO spectra for the three transition metal compounds **1**–**3**, where the respective spatial distributions of the frontier orbitals are shown as *insets*. The different symmetries of the involved metal *d* states are highlighted by the following *color code* *d* *xz* *red*, *d* *yz* *blue*, *d* *xy* *green*

The respective transmission functions as well as respective ground state MO spectra computed from non-equilibrium Greens functions calculations based on DFT (NEGF-DFT) [61, 62] in a junction environment and a subsequent subdiagonalization of the molecular subspace of the Hamiltonian matrix for the scattering region, respectively, are shown in Fig. 4 (further details about the definition of the scattering region and leads are given in “Methods”). It can be seen that the structural variation within the set of the three complexes has an impact on their coherent electron transport behavior, where for all three compounds the molecular eigenstates containing the *d*_*xz*_ and *d*_*yz*_ metal AO are defining the conductance, i.e., *T*(*E*) at *E* = *μ* (here and in the following the *z* direction will be chosen as the transport direction). The conductance value of **1**, being 0.078 *G*_0_, exceeds the ones of **2** and **3**, which are 0.012*G*_0_ and 0.032*G*_0_, respectively, where *G*_0_ = 2*e*^2^/*h* = 77.48 μS is the conductance quantum. This can be attributed to the higher degree of electronic coupling of its almost degenerate HOMO and HOMO-1 with the electrodes’ surface states, which we also list explicitly in Table 1. These differences in electronic coupling can be directly related to respective differences in the molecular structure. In **2** and **3**, the phenyl groups in transport direction reduce the electronic coupling by two factors: (1) in general, the amplitude of the MOs at the molecule–electrode interface is decreased as a consequence of increasing the size of the MOs, while maintaining their normalization; (2) the phenyl rings seem to also reduce the amount of conjugation over the bridge for **2** and **3**, which is particularly the case for the MO involving the metal AO with *d*_*yz*_ symmetry. In contrast to the *I*/*V* behavior of **1**, this reduction in electronic coupling makes a temporary localization of a hole in the HOMO-1 possible, which is crucial for explaining the reversible hysteresis for **2** found in the experiments performed in Ref. [1].

**Table 1:** Electronic coupling *H*_α,K_/eV of the molecular frontier orbitals to the electrodes as determined from Eq. (14)

	**1**	**2**	**3**
*d*_*xz*_	4.9 × 10^−2^	2.1 × 10^−2^	2.0 × 10^−2^
*d*_*yz*_	5.0 × 10^−2^	2.6 × 10^−3^	1.6 × 10^−3^
*d*_*xy*_	–	–	1.2 × 10^−5^

Here, we list and compare the MOs for all three compounds in terms of the involved metal center’s d-AO symmetries as they are also marked in the insets of Fig. 4

In contrast to the Fe containing compounds **1** and **2**, a triplet state has been determined as the ground state for compound **3**, which contains Mo. As a consequence only for **3**, a splitting of the eigenenergies is found for different spins, changing the energetic sequence of its MOs close to *μ* and only the MO containing the metal *d*_*xy*_ AO is occupied for both spins for this compound, making it now its HOMO.

For all compounds, the spatial distributions of the frontier orbitals, which are situated near the Fermi Level of the electrodes in a junction setup, are shown as insets in Fig. 4. It can be seen that the *d*_*xz*_ and *d*_*yz*_ metal AOs hybridize rather strongly with the respective ligands leading to a delocalization of the resulting MOs in the transport direction, whose contribution to the phase coherent conductance is dominant. The *d*_*xy*_ orbital, on the other side, is not oriented along the transport direction and is, therefore, not contributing to the coherent tunneling conductance. Its very low (but still finite) coupling to the metallic bands combined with its energetic proximity to *μ* in **3**, however, makes this MO accessible for electron hopping, which can cause reversible, but now also irreversible switching events in *I*/*V* measurements, as we discuss in the following.

By applying the two-channel model described earlier in this article, we were able to reproduce the key characteristics of the experimentally determined *I*/*V* curves by Schwarz et al. [1], namely pocket-like hysteresis features for **2** and both reversible and irreversible switching for **3**. In the following analysis, we would like to focus our attention on the irreversible switching events found for compound **3**.

**Fig. 5:**
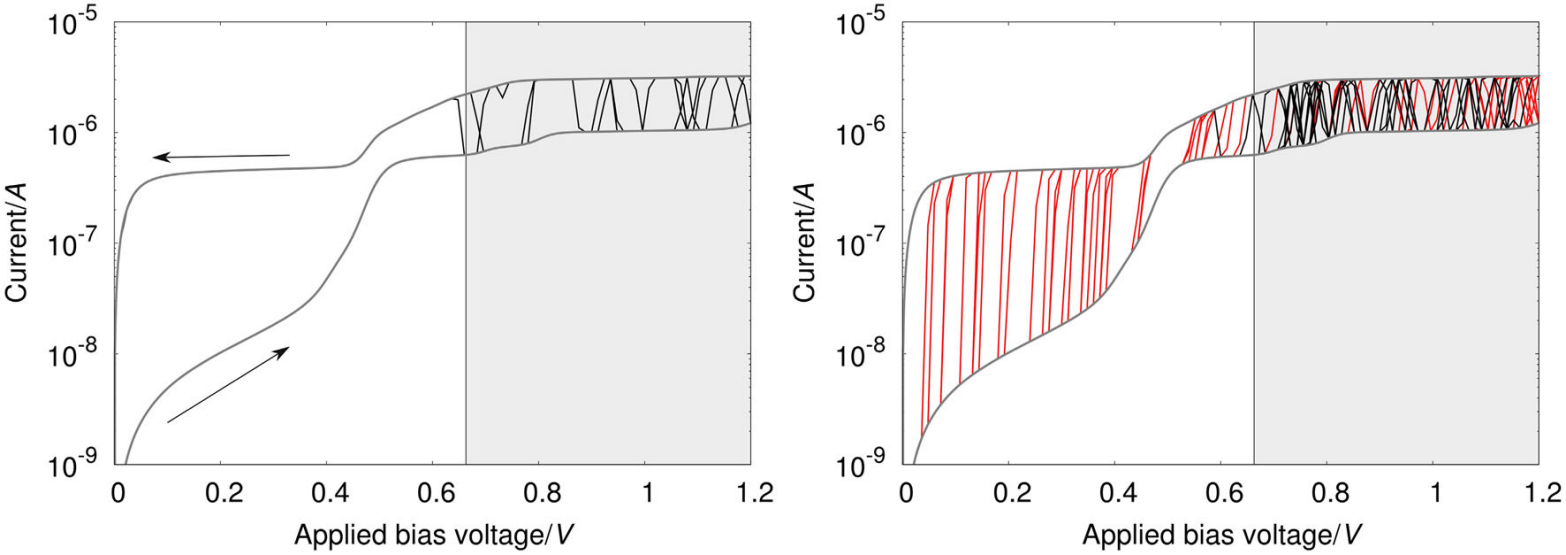
*I*/*V curves* resulting from 100 independent simulation runs obtained by applying the procedure from Eqs. (17) to (21) for a symmetric junction containing compound **3**, where the curves exhibiting irreversible (*left panel*) and reversible (*right panel*) conductance switching are shown separately. The switching events arising from an oxidation and reduction of the molecule are depicted in *black* and *red*, respectively. The *gray shaded* area represents the bias range, where *V* > *V*_crit,ox,R_. The parameters, which we calculated for compound **3** from DFT as described in the main text and used for this simulation, are: *T* = 50 K, Δ*G* ^0^ = 0.269 eV, *λ* = 62 meV, *H*_*α,*L_ = *H*_*α,*R_ = 1.2 × 10^−8^ eV, *V*_max_ = 1.2 V, *n*_V_ (number of bias steps in one direction) = 100, Δ*t* = 15 ms, *n* = 1000

The measurements performed by Schwarz et al., consisting of subsequent *I*/*V* sweeps on gradually stretched junctions in a MCBJ setup, have shown a reversible hysteretic behavior over a wide range of electrode–electrode distances. At high distances near the junctions rapture point, however, irreversible switching occurred. This situation was mimicked by our simulations as can be seen from the *I*/*V* curves shown in Fig. 5, which have been obtained by applying the procedure from Eqs. (17) to (21). The junction in this setup was modeled containing **3** symmetrically adsorbed to Au electrodes in top positions, regarding a single Au contact atom, where the Au–S distance was chosen to be 2.84 Å on each side, to simulate an idealized junction with the electrode-molecule distance near the junctions rapture point. Compared with the equilibrium situation for flat surfaces, where the molecule would be adsorbed in a hollow position with an Au–S distance of 2.34 Å, the determined electronic coupling in the top configuration is reduced by an order of magnitude. In order for irreversible conductance switching to occur for a symmetric system in our simulations, the resulting value for the electronic coupling would, however, still be too large. This situation changes when we also include a scaling factor of 1/100 to account for the idealized junction structure used in the simulation, where perfect symmetry and flat electrode surfaces are used. Such a surface model is not likely to mimic the electrodes in the actual MCBJ experiments, where they are created by breaking a direct Au–Au contact and atomic details of the resulting surface structures are unknown. Due to the nature of this experimental procedure, it is more likely that the molecule is adsorbed on rather corrugated parts of the electrode surfaces, where the tails of the surface states responsible for the electron coupling with the molecular eigenstates are distorted compared with a perfectly flat surface, thereby reducing the electronic coupling when moving from the idealized to the realistic. Furthermore, the self interaction error in DFT leads to an artificial delocalization of the MO containing the *d*_*xy*_ Mo-AO, thereby also artificially increasing the through space electronic coupling of this orbital to the leads.

In the left panel of Fig. 5, the *I*/*V* sweeps resulting in irreversible switching in our simulations are shown for the positive bias range. Such irreversible switching has been found in 16 out of 100 independent simulation runs. As can be seen from the figure, in which the system resides in the lower conducting (reduced) state at the start, an oxidation reaction can happen once *V*_crit,ox,r_ is reached, leading to a substantial increase in the conductance of the junction. In the selected curves, the reduction back into the ground state does not happen during the timespan of the simulation run, therefore, leaving the system in its charged state even when the bias is turned off again. As a consequence, on/off ratios of up to 200 can be achieved in these sweeps at small voltages. For 61 out of the 100 runs, on the other hand, after the oxidation of the compound into its charged state, a reduction back into its ground state happens during the respective simulation runs. This latter finding can be rationalized in terms of *k*_ox_ and *k*_rd_, as shown in Fig. 2. For the oxidation reaction to occur, the system needs an applied voltage which reduces the energetic barrier defined by Δ*G*^0^ and *λ*; therefore, this reaction is very unlikely before *V*_crit,ox,K_ is reached. For the reduction reaction, on the other side, *k*_rd_ does never fall below the preexponential *γ* defined in Eq. (3), since (at least) one of the electrodes always enables the reaction. Additionally, *k*_*rd*_ even reaches its maximum of *2γ* at biases in the range *V*_crit,rd,L_ < *V* < *V*_crit,rd,R_, making the reduction of the system into its ground system even more probable.

Since the microscopic structure in experimental MCBJ junctions is unknown, structural information regarding their symmetry can only be deduced from individual *I*/*V* traces, which are rarely found to be symmetric with respect to the current direction. Therefore, we also studied asymmetry in our simulated junctions by introducing a factor *H*_α,L_/*H*_α,R_. In terms of Fig. 2, such an asymmetry factor changes the relation between *k*_ox_ and *k*_rd_ in the way, that in the bias range studied in Fig. 5, *k*_ox_ is only dependent on *H*_α,R_, with *k*_ox,L_ negligible in the whole positive bias range. For *k*_rd_, however, the situation is different in the sense that at *V* < *V*_crit,rd,R_ both *k*_rd,L_ and *k*_rd,R_ are maximal, therefore, leading to *k*_rd_(*V* < *V*_crit,rd,R_) = *γ*_L_ + *γ*_R_. For biases above *V*_crit,rd,R_, on the other side, *k*_rd_,_R_ *→* *0*, leading to *k*_rd_, (*V* > *V*_crit,rd,R_) = *γ*_L_. In other words, this means that reducing the ratio *γ*_L_/*γ*_R_. = *(H*_α,L_/*H*_α,R_*)*^*2*^, while keeping *H*_DA,R_ constant does not influence the rate of the oxidation reaction, while the reduction probability is strongly reduced. This finding indicates that the probability for irreversible switching events to occur is systematically enhanced by structural asymmetry in the junction.

Numerical simulations demonstrating this effect of asymmetry in the molecule–electrode coupling on the corresponding number of occurrences of switching events are given in Table 2. As expected, the number of irreversible switching is increased systematically, when *H*_α,L_ is reduced relative to *H*_α,R_. The number of simulation runs, where no hopping events have been found and, however, does not change significantly because the first switching is always an oxidation and, therefore, solely depends on *k*_ox_, which is the same for all three cases. This finding explains, why irreversible switching was found reproducibly only for some samples in the measurements, where the shape of the electrodes and the atomic details of their contact to the molecule cannot be controlled in a MCBJ setup. Therefore, the asymmetry in the experimental junctions cannot be reliably reproduced with each investigated sample.

**Table 2:** Statistics of the switching behavior for **3**, where the ratio between the coupling to the left and right electrodes is varied systematically

*H*_DA,L_/*H*_DA,R_	Reversible switching	Irreversible switching	No switching
1	61	16	23
0.1	27	43	30
0.01	17	54	29

100 independent simulation runs have been performed for each value of the coupling ratio. *H*_α,R_ has been kept constant in all three simulation runs, while *H*_α,L_ has been reduced to arrive at the given ratios. The applied parameters for these simulation runs can be found in the caption of Fig. 5

In summary, we gave a detailed account of the theory behind the measured irreversible switching events reported recently. These events can be explained in terms of electron hopping onto a localized state of the compound near the electrodes Fermi level. The bias dependence of the reaction rates for both oxidation and reduction has been discussed in a junction environment applying a model based on DFT results with coherent electron tunneling for the conductance and electron hopping for the switching, which enables us to qualitatively reproduce the experimentally found behavior. Statistics over 100 simulation runs show that irreversible switching happens in around 16 % of the cases, while reversible switching due to a reduction of the system back into its ground state is dominant. The ratio between irreversible and reversible switching events can, however, be increased by introducing asymmetry in the junction, which is also likely to be encountered in the MCBJ experiments the simulations are mimicking.

## Methods

All electronic structure calculations in this paper were performed with the GPAW code [63, 64], in which the core electrons are described by the projector augmented wave (PAW) method and the basis set for the Kohn–Sham wave functions has been chosen to be a linear combination of atomic orbitals (LCAO) [65] on a double-zeta level with polarization functions (DZP) for all electronic structure calculations. The sampling of the potential energy term in the Hamiltonian is done on a real-space grid when using GPAW, for which we chose 0.18 Ǻ for its spacing and a Perdew–Burke–Ernzerhof (PBE) parametrization for the exchange–correlation (XC) functional throughout this paper. The scattering region for the NEGF-DFT scheme was defined by the molecular compound between two Au-fcc electrodes with 6 × 6 atoms in the surface plane in (111) orientation and one or three Au ad atoms for modeling top and hollow adsorption configurations, respectively. These rather large surfaces have been chosen for the purpose of excluding possible interactions of the molecule with its images in neighboring cells. All DFT calculations for such defined scattering regions were performed allowing for spin polarization and applying periodic boundary conditions, where seven layers of gold were used to reach Au bulk potential as required for the matching with the leads. The electronic structure for the lead regions has been obtained from Au bulk calculations with 6 × 6 × 3 Au atoms in the unit cell with a 1 × 1 × 15 k-point mesh, where the *z* direction was defining the transport direction.
